# The association between neonatal vitamin D status and risk of schizophrenia

**DOI:** 10.1038/s41598-018-35418-z

**Published:** 2018-12-06

**Authors:** Darryl W. Eyles, Maciej Trzaskowski, Anna A. E. Vinkhuyzen, Manuel Mattheisen, Sandra Meier, Helen Gooch, Victor Anggono, Xiaoying Cui, Men Chee Tan, Thomas H. J. Burne, Se Eun Jang, David Kvaskoff, David M. Hougaard, Bent Nørgaard-Pedersen, Arieh Cohen, Esben Agerbo, Carsten B. Pedersen, Anders D. Børglum, Ole Mors, Pankaj Sah, Naomi R. Wray, Preben B. Mortensen, John J. McGrath

**Affiliations:** 1 Queensland Brain Institute, University of Queensland, St. Lucia, Queensland, Australia; 2 Queensland Centre for Mental Health Research, The Park Centre for Mental Health, Wacol, Queensland, Australia; 3 Institute for Molecular Bioscience, The University of Queensland, Brisbane Queensland, Australia; 4 Department of Biomedicine, Aarhus University, Aarhus, Denmark; 5 iPSYCH, The Lundbeck Foundation Initiative for Integrative Psychiatric Research, Aarhus, Denmark; 6 Mental Health Centre for Child and Adolescent Psychiatry, Copenhagen Region, Copenhagen, Denmark; 7 Clem Jones Centre for Ageing Dementia Research, University of Queensland, St. Lucia, Australia; 8 Center for Neonatal Screening, Department for Congenital Disorders, Statens Serum Institut, Copenhagen, Denmark; 9 National Centre for Register-Based Research, Aarhus University, Aarhus, Denmark; 10 Centre for Integrated Register-based Research, Aarhus University, Aarhus, Denmark; 11 Department of Biomedicine and iSEQ-Centre for Integrative Sequencing, Aarhus University, Aarhus, Denmark; 12 Psychosis Research Unit, Aarhus University Hospital, Psychiatry, Aarhus, Denmark

## Abstract

Clues from the epidemiology of schizophrenia, such as the increased risk in those born in winter/spring, have led to the hypothesis that prenatal vitamin D deficiency may increase the risk of later schizophrenia. We wish to explore this hypothesis in a large Danish case-control study (n = 2602). The concentration of 25 hydroxyvitamin D (25OHD) was assessed from neonatal dried blood samples. Incidence rate ratios (IRR) were calculated when examined for quintiles of 25OHD concentration. In addition, we examined statistical models that combined 25OHD concentration and the schizophrenia polygenic risk score (PRS) in a sample that combined the new sample with a previous study (total n = 3464; samples assayed and genotyped between 2008-2013). Compared to the reference (fourth) quintile, those in the lowest quintile (<20.4 nmol/L) had a significantly increased risk of schizophrenia (IRR = 1.44, 95%CI: 1.12–1.85). None of the other quintile comparisons were significantly different. There was no significant interaction between 25OHD and the PRS. Neonatal vitamin D deficiency was associated with an increased risk for schizophrenia in later life. These findings could have important public health implications related to the primary prevention of schizophrenia.

## Introduction

Schizophrenia is a poorly understood group of brain disorders characterised by impairments in cognition, perception and affect, with a lifetime prevalence of 0.7%^1^. As with other heterogeneous disorders, the expectation is that schizophrenia is associated with many different causal factors involving both common and rare genetic variants, as well as a range of environmental exposures. With respect to environmental risk factors for schizophrenia, much attention has focussed on modifiable prenatal and early life exposures^2^. Epidemiologic research has identified an increased risk of schizophrenia associated with winter/spring season of birth^3^, living in high latitude settings^4^, early life urban residence^5^ and migrant status^6^ (especially dark-skinned migrants to high latitude countries). Vitamin D status maps onto these same risk factors since the prevalence of vitamin D deficiency is higher in (a) winter/spring versus summer/autumn, (b) high versus low latitude settings, (c) urban versus rural settings, and (d) dark- versus light-skinned ethnic groups (especially prominent in high latitude countries)^7^. In light of these clues, developmental vitamin D deficiency has been proposed as a candidate risk factor for schizophrenia^8^.

Hypotheses linking developmental vitamin D deficiency and altered brain development are biologically plausible, since the vitamin D receptor (VDR) is expressed in the brain, particularly in areas of interest to schizophrenia research such as dopaminergic-rich brain regions^9^. Animal experiments have also provided robust evidence that developmental (i.e. transient prenatal) vitamin D deficiency is associated with a range of persistent neurochemical and behavioural outcomes of interest to neuropsychiatric research^10^. Furthermore, a schizophrenia case-control study (n = 848) demonstrated that neonates with vitamin D deficiency had an increased risk of being diagnosed with schizophrenia in later life (i.e. lowest versus reference [fourth] quintile Incidence Rate Ratio (IRR) = 2.1; 95% CIs 1.3–3.5)^11^. Unexpectedly, this initial study also found a non-linear exposure-risk relationship, with neonates in the highest quintile of the distribution having an increased risk (IRR = 1.71; 95%CI, 1.04–2.8). We had the opportunity to explore the relationship between neonatal vitamin D status and the risk of schizophrenia in a new and larger Danish case-control sample. We predicted that neonatal vitamin D deficiency would be associated with an increased risk of schizophrenia.

In addition to the assessment of vitamin D status, we explored statistical models that combined both neonatal vitamin D status and schizophrenia Polygenic Risk Scores (PRS). The presence of such interactions can have important implications from a public health perspective. For example, for environmental risk factors with a significant gene by environment interaction, preventive interventions designed to reduce exposure to the environmental risk factor may be better targeted to subgroups of the population with the susceptible genetic risk factors. Conversely, the absence of significant gene by environment interaction may lend weight to more widespread (universal) preventive interventions. While gene by environment interaction studies require large sample sizes (compared to the samples required to detect the main effects of the genetic and environmental risk factors)^12^, we had the opportunity to explore these research questions based on Danish neonatal samples that had been both assayed for 25OHD concentration and genotyped. To date, psychiatric research based on large case-control samples with both genetic and environmental risk factors has been scant^13^.

## Method

### Case-control study

We studied participants from the Danish national registry, which is based on record linkage between the Danish Psychiatric Central Register, the Danish Civil Registration System^14^ and the Danish Newborn Screening Biobank^15^. Newborn dried blood spots have been systematically collected and stored since May 1, 1981 and stored at −20 degrees C. Cases were randomly selected from all those born in Denmark between 1981–2000 who received a diagnosis of schizophrenia according to International Statistical Classification of Diseases, 10^th^ Revision^16^ code F20. Most of these cases developed schizophrenia between September 2005 and December 2008. Controls, drawn from the same birth cohort, were individually matched on sex and date of birth, and were alive and free of schizophrenia at the time of onset of the matched case. The Danish Data Protection Agency and the ethics committees of the Central Denmark Region approved this study, and all methods were performed in accordance with relevant guidelines and regulations.

#### Assessment of vitamin D status

3.2 mm DBS (samples and calibrants) were hydrated by the addition of 50 µL water and shaken for 30 min at room temperature. Vitamin D species were extracted with acetonitrile (500 µL) containing *6,19,19*-[^2^H_3_]-25OHD_2_ and *6,19,19*-[^2^H_3_]-25OHD_3_ (0.5 pmol) as internal standards. After mixing, supernatants (500 µL) were subjected to solid-phase ion exchange using zirconium dioxide (ZrO_2_) and titanium dioxide (TiO_2_) (Glygen, USA) to remove ion-suppressing phospholipids. Eluted samples were evaporated to dryness then derivatised with 4-phenyl-1,2,4-triazioline-3,5-dione (PTAD), (50 µL, 0.1 mg/mL in anhydrous ethyl acetate). After 30 min samples were again evaporated to dryness.

Samples were reconstituted in 33% acetonitrile/water (60 µL) on a plate shaker at room temperature (650 rpm, 1 min). Samples were injected onto a Kinetex column XB-C18, 50 × 2.1 mm, 1.5 µm (Phenomenex, CA, USA) by a peak-focusing technique (30 µL sample injection volume +20 µL water from an open 2-mL glass vial) using an integrated pretreatment program. The LC-MS/MS system consisted of a UHPLC (Nexera X2, Shimadzu Ltd, Japan), comprising a SIL-30AC autosampler (50 µL loop), two LC-30AD binary pumps, DGU-20A5 degasser, and CTO-30A oven, and interfaced to a 5500 QTRAP mass spectrometer (SCIEX, Canada) with Atmospheric Pressure Chemical Ionisation (APCI) TurboIonSpray ion source. Reference samples for neonatal dried blood are not available. Therefore we prepared an “in house” dried blood spot sample for inter-assay variance. The inter-assay variation over 2 years was 11.6%^17^. The sera calibrators supplied by the National Institute of Standards and Technology (NIST 1950) were used in each plate. Using these external calibrants, overall assay inaccuracy was <4%. Previous studies have demonstrated a strong correlation between this dried blood spot assay and cord sera 25OHD concentration (r = 0.85, P < 0.0001)^18^. Full details of the assay are described elsewhere^17^.

Total 25OHD was reported as the sum of 25-hydroxyvitamin D_2_ (25OHD_2_) and 25-hydroxyvitamin D_3_ (25OHD_3_) species as previously described, and the inter- and intra-assay coefficients of variability were 6.9% and 11.6% respectively^17^.

### Genotyping and generation of the PRS

The genotyping and quality control details for this sample (henceforth referred to as DK2016) have been published previously^19^. In summary, DNA was extracted from the neonatal dried blood spots stored at the Statens Serum Institut, whole-genome amplified in triplicate using the Qiagen REPLI-g mini kit [Qiagen, Hilden, Germany] (the three separate reactions were pooled), and then genotyped with Illumina Human 610-Quad BeadChip array (DK2016a) or Illumina Infinium CoreExome beadchip (DK2016b)(Illumina, San Diego, CA). In order to maximizes sample size, we combined our new DK2016 sample with a previously published independent case-control sample (henceforth referred to as DK2010)^11^. The original samples for DK2010 were 423 cases and 425 controls. The original samples for DK2016a and DK2016b were 387 cases and 391 controls, and 923 cases and 924 controls respectively. 8 subjects were removed for ambiguous sex (genotype versus register values) and 1 subject was removed for a very high concentration of 25OHD_2_. The models that examined the association of 25OHD on risk of schizophrenia was restricted to matched pairs, which are a subset of these samples.

We generated PRS in our Danish sample using allelic effect sizes estimated in the latest GWAS for schizophrenia from the Schizophrenia Working Group of the Psychiatric Genomics Consortium^20^. The Danish samples were excluded from this ‘discovery sample’, leaving a sample of 34,600 cases and 45,968 controls. Specifically, we selected single nucleotide polymorphisms (SNPs) that were present in both our combined dataset and the PGC data. SNPs were also filtered on PLINK imputation INFO parameter retaining those with values > 0.8 in both data sets. Next, SNPs were pruned based on linkage disequilibrium (LD) r^2^ (r^2^ > 0.2 in 500 kb window) using the clumping function in PLINK 1.07^21^ (i.e. preferentially retaining the most associated SNPs in an LD regions), and we excluded SNPs in the extended Major Histocompatibility Complex (MHC) region (chr6:25–34 Mb; except retaining rs7746199). We created a single PRS for each individual based on SNPs with p-value < 0.05; this decision was justified as the threshold that maximised variance explained in leave-one-out analyses^20^. These algorithms were applied to datasets within each genomic array and the resultant PRSs were standardised within each chip and sex and then merged.

### Statistical analyses

In keeping with our previous analyses of neonatal blood samples^11^, we derived quintiles for 25OHD based on the control sample, and used conditional logistic regression (Incidence Rate Ratio; IRR, and 95% confidence intervals) to assess the relationship between neonatal 25OHD concentration and risk of schizophrenia. As our previous study identified a non-linear relationship (both the lowest and highest quintiles were associated with increased risks of schizophrenia compared to the fourth quintile), we prespecified the fourth quartile as the reference category. Based on previous research using the same psychiatric case register and based on factors known to be associated with vitamin D status^22^ we explored the influence of a range of variables on the association between the variables of interest (using likelihood ratio tests). These include maternal, paternal, and sibling history of any mental disorder, sex, age second-generation immigrant status, degree of urbanization at place of birth, maternal and paternal age at the time of the child’s birth, gestational age, birth weight, and birth length. Because the cases and controls were individually matched on sex, date of birth/age, all relative risks were controlled for these variables. Population-attributable fraction was calculated according to the recommendations of Bruzzi *et al*.^23^ (equation 10).

For the analyses that examined both 25OHD concentration and PRS scores in the combined samples, the 25OHD concentrations were first standardised within the assay runs (DK2010 and DK2016) and transformed using Van der Waerden-normalised scores^24^. The range and distribution of 25OHD concentrations were similar between the two samples. A sample of QC-ed and LD-pruned SNPs (approximately 40,000) were used to calculate principal components (PCs) in PLINK 1.9^25^ and ancestral outliers mean +/−4 SD for each of the first two PCs (loss of 414 individuals giving a total sample of 3129). After excluding these outliers, PC axes were recalculated. None of the PC axes were associated with schizophrenia status (up to 10 PCs tested). However, PC1 was significantly associated with both PRS and 25OHD concentration.

Based on the combined sample, we evaluated main and interaction effects of our two predictors (adjusted for PRS and 25OHD) using PC1-controlled residuals to adjust for population stratification (PRS_res_, vitD_res_, respectively). We examined the influence of 25OHD as quintiles and as a continuous variable. We explored gene-environment interactions (GxE) with the logistic regression, Y~ PRS_res_ + vitD_res_ + vitD_res_*PRS_res_. Levels of significance were tested using Likelihood Ratio Test (LRT) comparing −2loglikelihood fit statistic of these alternative models to the fit of the null model (Y~1 + E). In line with the previous publication^11^, we also investigated GxE using conditional logistic regression where PRS scores were split into quintiles of 25-hydroxyvitamin D (Y~ PRS_res_.vitDq1 + PRS_res_.vitDq2 + PRS_res_.vitDq3 + PRS_res_.vitDq3 + PRS_res_.vitDq4 + PRS_res_.vitDq5 + Q + E); the 4^th^ quintile was used as a reference. Where PRS_res_.vitDq1–5 are the PRS for those individuals in each of the 25OHD quintiles, Q is a covariate indexing the quintiles and E is a residual. The interaction was tested using LRT test where comparing the full model to the reduced model Y~PRS_res_ + Q + E. We also assessed models based on matched case-controls (i.e. the analyses were stratified on case-control pairs). However, this produced virtually identical results (not shown). We confirmed that including the top 20 PC axes had no impact on results. In order to quantify the variance explained by 25OHD, the PRS and their interaction, we calculated Naglekerke’s R^2^ and transformed the odds ratios to R^2^ on the liability scale^26^. The latter transformation incorporated the prevalence of schizophrenia in the general community, as well as the prevalence in the current sample, in order to adjust for oversampling in the case-control sample.

## Results

### Correlates of 25OHD concentration in the new sample

**Figure 1:**
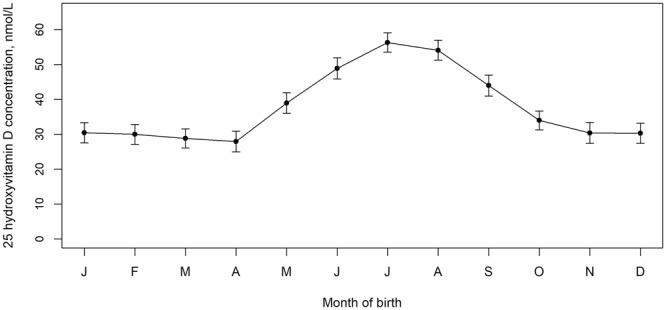
Mean monthly 25 hydroxyvitamin D in nmol/L (95% confidence intervals). Note the characteristic seasonal variation, with lower 25 hydroxyvitamin D concentrations in winter and spring born infants (coincident with seasons of increased risk of schizophrenia).

The demographic characteristics of the sample and the distribution of 25OHD by quintile are shown in Table 1. Consistent with the epidemiology of vitamin D deficiency, we confirmed that mean monthly 25OHD concentrations displayed the expected winter/spring nadir (Fig. 1). Furthermore, 25OHD concentration varied by parents place of birth. The mean (95% confidence intervals) for 25OHD subjects with both parents born in Denmark was 38.3 (37.3–39.3) nmol/L, while those with one or both parents born outside of Denmark had lower 25OHD concentrations (33.9, 31.2–36.6 nmol/L).

**Table 1:** Characteristics of the 1301 cases and 1301 controls.

	Percentage of cases	Number of cases	Percentage of controls	Number of controls
Total		1301		1301
Quantile of vit D				
<20.4	26.1	339	20.2	263
20.4-29.8	19.8	258	19.8	258
29.9-40.0	16.5	215	20.1	261
40.1-53.5	18.3	238	19.9	259
> = 53.6	19.3	251	20.0	260
Gender				
Male	56.1	730	56.1	730
Female	43.9	571	43.9	571
Immigration status				
Second gen. immigrant	15.3	199	9.3	121
Native Dane	83.1	1081	89.6	1166
Unknown	1.6	21	1.1	14
Degree of urbanization				
Capital	15.1	196	11.7	152
Capital suburb	14.8	192	14.3	186
Provincial cities	11.0	143	11.7	152
Provincial towns	30.0	390	26.6	346
Rural areas	29.2	380	35.7	465
Maternal age at child’s birth, years				
12–19	5.8	76	3.3	43
20–24	28.1	365	25.1	326
25–29	36.3	472	39.8	518
30–34	21.5	280	22.5	293
35 and above	8.3	108	9.3	121
Paternal age at child’s birth, years				
12–19	1.2	16	0.8	10
20–24	16.2	211	13.7	178
25–29	30.5	397	33.1	430
30–34	28.9	376	30.8	401
35–39	13.6	177	14.8	193
40 and above	8.2	107	5.9	77
Unknown	1.3	17	0.9	12
Gestational age, completed weeks				
25–36	5.8	75	5.1	66
37–39	32.9	428	32.1	418
40 and above	60.1	782	62.2	809
Unknown	1.2	16	0.6	8
Birth Weight, grams				
Below 2500	6.8	88	5.0	65
2500–2999	15.9	207	12.2	159
3000–3499	32.4	421	33.5	436
3500–3999	30.2	393	33.7	439
4000 and above	14.1	183	15.3	199
Unknown	0.7	9	0.2	3
Birth length, cm				
35–49	20.1	261	15.3	199
50–51	28.3	368	31.1	405
52–54	40.0	520	41.8	544
55 and above	10.0	130	10.5	136
Unknown	1.7	22	1.3	17
Maternal mental illness				
History	19.6	255	8.5	111
No history	80.4	1046	91.5	1190
Paternal mental illness				
History	17.6	229	6.9	90
No history	82.4	1072	93.1	1211
Sibling mental illness				
History	17.4	227	9.2	120
No history	82.6	1074	90.8	1181

### Neonatal 25OHD and risk of schizophrenia in the new sample

**Figure 2:**
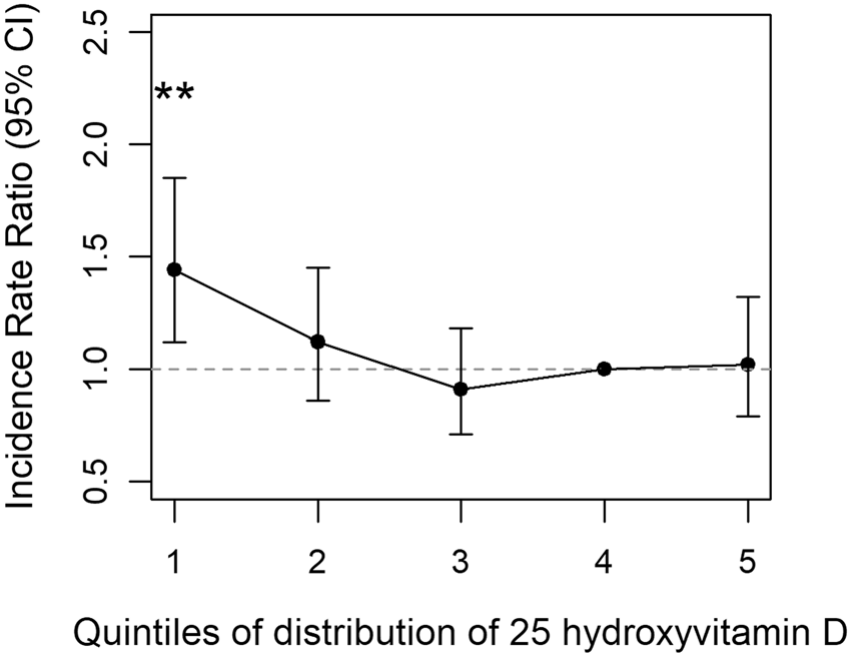
Incidence Rate Ratio and 95% confidence intervals for schizophrenia by quintiles of 25 hydroxyvitamin D concentration, a nested case-control study of 1301 cases and 1301 controls. There was a significantly increased Incidence Rate Ratio for those in the lowest quintile versus the fourth (reference) quintile (IRR = 1.44, 95% CI: 1.12–1.85, p = 0.004). None of the other comparisons were statistically significant.

Based on the distribution of 25OHD in the control sample, the quintile cuts for the 20^th^, 40^th^, 60^th^ and 80^th^ percentiles were 20.4, 29.9, 40.1, and 53.6 nmol/L respectively. Compared to the reference category (fourth quintile), those in the lowest quintile had an increased risk of schizophrenia (IRR = 1.44, 95% CI: 1.12–1.85, p = 0.004) (Fig. 2). None of the other comparisons were statistically significant (IRR and 95%CI 1.12, 0.86–1.45; 0.91, 0.70–1.18; 1.02, 0.79–1.32 for second, third and fifth quintiles versus reference quintile, respectively). This general pattern of findings persisted in models adjusted for family history of any mental disorder, sex, age, degree of urbanization at place of birth, maternal and paternal age at the time of the child’s birth, gestational age, birth weight, or birth length (data not shown), however when stratified by second-generation immigrant status there was significant difference in model fit (p = 0.04). The general pattern of finding was identified in the native Danes (lowest versus fourth quintile IRR = 1.69, 95%CI: 1.24–2.15), but not in second generation migrants (none of the comparisons were significant - lowest versus fourth quintile IRR = 1.01, 95%CI: 0.77–1.33). With respect to the population attributable fraction, optimizing neonatal 25OHD status (i.e. shifting the population to optimal levels within the reference category) could account for 8.4% of the incidence of schizophrenia in this setting.

### The influence of neonatal 25OHD and PRS and risk of schizophrenia in the combined samples

Based on the combined sample, both the lowest and second lowest 25OHD quintiles were associated with an increased risk of schizophrenia compared to the reference fourth quintile (IRR = 1.52, 95% CIs 1.20–1.93; IRR = 1.31, 95% CI 1.03–1.67 respectively). None of the other comparisons were statistically significant (1.16, 0.92–1.46; 1.15, 0.91–1.46; for third and fifth quintiles versus reference quintile, respectively). Main effect analyses confirmed the influence of (a) PRS on schizophrenia risk (IRR = 1.33, 95% CI 1.24–1.43; higher PRS associated with higher risk) and (b) 25OHD concentration on schizophrenia risk when included as a continuous variable (IRR = 0.92, 95% CI 0.86–0.99; higher 25OHD associated with lower risk). When modelled together in logistic regression, PRS and 25OHD concentration produced nearly identical IRRs to those acquired from their individual models (IRR = 1.4, 95% CI 1.25–1.44; IRR = 0.91, 95% CI 0.85–0.98 respectively). There was no evidence of a significant interaction between PRS and 25OHD (p = 0.33). On the liability scale the PRS and 25OHD explained ~1% and ~0.09% of the variance respectively (Naglekerke’s R2 0.03 and ~0.002 respectively). When combined, both explained 1.2% (SE = 0.003) of the variance on the liability scale (Naglekerke’s R2 0.03), in line with the expectations of an additive model. While not statistically significant, the interaction term explained 0.02% on the liability scale (Naglekerke’s R2 ~0.0004). Power analyses indicated that we had 80% power to detect 0.2% of the variance explained, but were underpowered to detect interaction effects less than that. Statistical models based on 25OHD quintiles and PRS also found no evidence of an interaction (data not shown).

## Discussion

We have confirmed that neonatal vitamin D deficiency was associated with a significantly increased risk of schizophrenia. Those with 25OHD below 20.4 nmol/L (consistent with standard definitions of vitamin D deficiency^27^), had a 44% increased risk of schizophrenia compared to those in the reference category. In keeping with our guiding hypothesis, we identified seasonal fluctuations in 25OHD concentrations (lowest in winter/spring) and lower concentrations in the offspring of migrants. As the developing fetus is totally reliant on maternal vitamin D stores, and neonatal 25OHD concentrations are strongly correlated with maternal sera concentrations at birth (r ~ 0.80)^18^, our findings support the hypothesis that maternal vitamin D deficiency is a risk factor for schizophrenia in the offspring.

While our previous study^11^ identified a U-shaped relationship between 25OHD and risk of schizophrenia, the current study identified that only those in the lowest quintile had an increased risk of schizophrenia. We note that the current study identified effect sizes for each of the quintile comparisons that were within the 95% confidence intervals reported in our previous study. With the greater precision afforded by the larger sample size (2602 in DK2016 versus 848 in DK2010), we would have had sufficient power to detect an association between higher concentrations of 25OHD and risk of schizophrenia had this reflected the nature of the exposure-risk relationship. While the evidence linking neonatal vitamin D deficiency and an increased risk of schizophrenia has been replicated, the evidence with respect to higher vitamin D concentrations and risk of schizophrenia remains mixed. From a public health perspective, there is a growing consensus that the use of vitamin D supplements should only target people at risk of deficiency, and that those with adequate 25OHD concentrations will not benefit from supra-normal concentrations^28^. We also found that the association between neonatal vitamin D deficiency and increased risk of schizophrenia was restricted to ethnic Danes, not second generation migrants (i.e. with at least one parent born overseas). We plan to explore this issue in future studies that have (a) a larger sample size of migrants, (b) additional case-control matching on migrant status (in addition to sex and date of birth), and (c) quintile determination based on second-generation migrants (versus the entire control population).

With respect to the specificity between neonatal vitamin D and risk of schizophrenia, recent evidence suggests that this exposure may also be associated with other neurodevelopmental disorders. For example, recent studies based on the Generation R birth cohort found that mid-gestational vitamin D deficiency (~21 weeks gestation) was associated with an increased risk of autism-related traits^29^ and autism-spectrum disorder^30^. These findings are in keeping with the general recognition that both genetic and non-genetic risk factors can be shared across different psychiatric phenotypes^31^. Thus, it is feasible that optimizing prenatal vitamin D status may impact on a broader range of mental health outcomes. Our findings also lend weight to the body of evidence implicating adverse prenatal nutrition with risk of schizophrenia^32^, and the potential for prenatal supplementation to lower the risk of neurodevelopmental disorders^33^.

In addition, we have examined how neonatal 25OHD concentrations influences risk when combined with PRS^13^. We found no evidence of an interaction between the PRS and 25OHD in our study. In light of the substantial polygenicity associated with schizophrenia and the very small variance explained by individual loci, it is clear that the field must await much larger sample sizes in order to confidently exclude small to moderate size gene-by-environment interactions. While the field expects that gene-by-environment interactions are important for mental disorders, the empirical evidence for these interactions currently remain scant^12,34^.

With respect to potential mechanisms of action linking developmental vitamin D deficiency and altered brain development, animal studies have demonstrated that transient prenatal vitamin D deficiency alters a range of outcomes related to brain volume, neurochemistry, gene and protein expression, and behavior^35^. It has been demonstrated that developmental vitamin D deficiency alters the specification and maturation of dopaminergic systems^36^. Furthermore, recent evidence from genetics has found that variants in the voltage-gated calcium channels are associated with risk of schizophrenia^37^. The active form of vitamin D is known to rapidly enhance activity of these channels in a range of tissues, including the brain^38–40^. As it is unusual that discoveries from epidemiology and genetics converge on shared neurobiological pathways, these mechanism warrant closer scrutiny in future studies.

In the absence of observed 25OHD concentration, it is also feasible to predict variance in vitamin D status based on the results of GWAS studies examining the genetic architecture of 25OHD. One study used a PRS based on four single nucleotide polymorphisms associated with 25OHD concentration^41^. This study found no evidence to support the hypothesis that scores linked to lower 25OHD concentration also predicted an increased risk of schizophrenia. However, based on clues from epidemiology, the critical window of exposure is during early development (prenatal and/or neonatal versus the entire lifespan). For prenatal exposures, Mendelian randomization may require both maternal and neonatal genotypes in order to accurately predict prenatal exposures.

While the sample size of this case-control study is one of the largest of its kind, the study has several limitations. The diagnosis of schizophrenia was based on Danish psychiatric registers rather than structured research instruments, however studies have found a high validity of schizophrenia diagnosis using these registers^42^. We only had one measure of 25OHD taken from neonatal blood, and thus it is feasible that there are other periods during gestation and/or early life when vitamin D deficiency may also influence risk of schizophrenia. While we assessed 25OHD status in newborn infants, it is feasible that the critical window for exposure remains open in the first year of life. For example, a study based on the Northern Finnish Birth Cohort found that lack of vitamin D supplements in the first year of life was associated with an increased risk of schizophrenia (by age 31 years) in males^43^. Individuals from non-Western countries (i.e. more likely to be dark-skinned) who migrated to the Netherlands between birth and age 4 years had elevated risk of later psychotic disorders (compared to those who migrated at older ages, and compared to native Dutch)^44^. Combined, these findings suggest that early post-natal life may also be a period of risk for exposure to vitamin D deficiency. If exposure to vitamin D deficiency during the first few years of post-natal life influences the subsequent risk of schizophrenia (separately or in addition to the putative risk associated with prenatal and neonatal deficiency), then routine neonatal screening of all neonates for vitamin D deficiency (plus the targeted use of supplements where needed), may be able to avert later adverse health outcomes. While our prior hypothesis implicated vitamin D deficiency as the putative causal agent, it is feasible that unmeasured factors may confound the association between vitamin D deficiency and risk of schizophrenia (e.g. maternal behaviours that lead to both vitamin D deficiency and exposure to other risk factors).

With respect to the now replicated association between neonatal vitamin D deficiency and risk of schizophrenia, randomized controlled trials of vitamin D supplements in pregnant women will be required to make confident public health recommendations^45^. These findings raise the tantalizing prospect that optimization of maternal vitamin D status may result in the primary prevention of schizophrenia in a manner comparable to the role of folate supplementation in the prevention of spina bifida.

## Data Availability

The extracts from the Danish health registers and the genotype data used in this study are not publicly available due to restrictions imposed by Danish Data Protection Agency and the governing ethics committees. Researchers who wish to access summary-level data should contact the corresponding author.
